# Disease-Related Cardiac Troponins Alter Thin Filament Ca^2+^ Association and Dissociation Rates

**DOI:** 10.1371/journal.pone.0038259

**Published:** 2012-06-04

**Authors:** Bin Liu, Svetlana B. Tikunova, Kristopher P. Kline, Jalal K. Siddiqui, Jonathan P. Davis

**Affiliations:** 1 Department of Physiology and Cell Biology, The Ohio State University, Columbus, Ohio, United States of America; 2 Department of Pharmacological and Pharmaceutical Sciences, University of Houston, Houston, Texas, United States of America; National Institute for Medical Research, Medical Research Council, United Kingdom

## Abstract

The contractile response of the heart can be altered by disease-related protein modifications to numerous contractile proteins. By utilizing an IAANS labeled fluorescent troponin C, 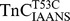, we examined the effects of ten disease-related troponin modifications on the Ca^2+^ binding properties of the troponin complex and the reconstituted thin filament. The selected modifications are associated with a broad range of cardiac diseases: three subtypes of familial cardiomyopathies (dilated, hypertrophic and restrictive) and ischemia-reperfusion injury. Consistent with previous studies, the majority of the protein modifications had no effect on the Ca^2+^ binding properties of the isolated troponin complex. However, when incorporated into the thin filament, dilated cardiomyopathy mutations desensitized (up to 3.3-fold), while hypertrophic and restrictive cardiomyopathy mutations, and ischemia-induced truncation of troponin I, sensitized the thin filament to Ca^2+^ (up to 6.3-fold). Kinetically, the dilated cardiomyopathy mutations increased the rate of Ca^2+^ dissociation from the thin filament (up to 2.5-fold), while the hypertrophic and restrictive cardiomyopathy mutations, and the ischemia-induced truncation of troponin I decreased the rate (up to 2-fold). The protein modifications also increased (up to 5.4-fold) or decreased (up to 2.5-fold) the apparent rate of Ca^2+^ association to the thin filament. Thus, the disease-related protein modifications alter Ca^2+^ binding by influencing both the association and dissociation rates of thin filament Ca^2+^ exchange. These alterations in Ca^2+^ exchange kinetics influenced the response of the thin filament to artificial Ca^2+^ transients generated in a stopped-flow apparatus. Troponin C may act as a hub, sensing physiological and pathological stimuli to modulate the Ca^2+^-binding properties of the thin filament and influence the contractile performance of the heart.

## Introduction

The heart is a highly dynamic organ that can regulate both its contractile strength and speed to accommodate the demands of the body. Numerous cardiac diseases adversely alter the heart’s ability to properly maintain its performance [1]. It is well established that aberrant intracellular Ca^2+^ signaling is associated with systolic and diastolic cardiac dysfunctions [2], [3]. Cardiac diseases may also alter how the heart responds to the Ca^2+^ signal [4]. For instance, numerous reports have demonstrated that myofilament Ca^2+^ sensitivity is affected by cardiomyopathy associated protein mutations, truncations and post-translational modifications [5], [6], [7], [8]. Thus, contractile dysfunctions may be caused by both altered intracellular Ca^2+^ signaling and abnormal myofilament responsiveness to the Ca^2+^ signal [4], [9].

Troponin C (TnC) is the myofilament Ca^2+^ sensor in cardiac muscle responsible for translating the intracellular Ca^2+^ signal into mechanical force [10]. The Ca^2+^ sensitivity of TnC can be modulated by multiple factors, including its interactions with other myofilament proteins, post-translational modifications of the myofilament, as well as cardiac disease-related protein modifications [11], [12], [13], [14]. In this regard, TnC is not just a passive element that transmits the Ca^2+^ signal. Instead, it may act as a central hub that integrates information from the myofilament (beneficial or maligned) and adjusts its Ca^2+^ binding properties to regulate cardiac muscle mechanics [15].

While it is clear that myofilament disease-related protein modifications can alter the steady-state Ca^2+^ sensitivity of TnC, much less is known regarding their effects on TnC’s Ca^2+^ exchange kinetics [16], [17], [18]. The kinetics of Ca^2+^ exchange with TnC may be even more significant to how the heart performs since the heart is dynamic and does not function in a static steady-state. Furthermore, it is the kinetics of Ca^2+^ exchange with TnC, including the rate of Ca^2+^ association to and dissociation from TnC that determine its steady state Ca^2+^ sensitivity. The rate of Ca^2+^ association is 3 to 4 orders of magnitude faster than the rate of Ca^2+^ dissociation, and considered diffusion controlled (for review see [15]). Thus, it is generally assumed that changes in the steady-state Ca^2+^ sensitivity of TnC are caused exclusively by modulating the rate of Ca^2+^ dissociation. However, our previous studies suggest the rate of Ca^2+^ association to TnC can also be altered [19], [20].

In this work, disease-related protein modifications of troponin I (TnI) and troponin T (TnT) were selected to systematically study their effects on Ca^2+^ binding and exchange with the troponin complex (Tn) and the thin filament using a fluorescently labeled TnC. The protein modifications include five dilated cardiomyopathy (DCM) mutations (TnI K36Q, TnT R141W, TnT R131W, TnT R205L, and TnT ΔK210), two hypertrophic cardiomyopathy (HCM) mutations (TnI S166F and TnT R92Q), two restrictive cardiomyopathy (RCM) mutations (TnI D190H and TnI R192H), and an ischemia-induced truncation of TnI (residues 1-192). These protein modifications represent a broad spectrum of diseases that change the steady-state Ca^2+^ sensitivity of the actomyosin ATPase activity and/or force generation [12], [13], [21], [22]. More importantly, some of these protein modifications also alter the kinetics and amplitude of cardiac muscle contraction and/or relaxation [23], [24]. We demonstrate that these disease associated protein modifications adversely altered steady-state Ca^2+^ binding to TnC by influencing both its Ca^2+^ association and dissociation rates on the thin filament (with almost no effect when measured in the isolated Tn complex). These results suggest that TnC acts as a central hub on the thin filament by sensing pathological stimuli to alter cardiac contractile properties.

## Materials and Methods

### Materials

Phenyl-Sepharose CL-4B, Tween-20, and EGTA were purchased from Sigma Chemical Co. (St. Louis, MO). IAANS and phalloidin were purchased from Invitrogen (Carlsbad, CA). Affi-Gel 15 affinity media was purchased from Bio-Rad (Hercules, CA).

### Protein Mutagenesis

The pET3a plasmid encoding human cardiac TnC was a generous gift from Dr. Lawrence Smillie (University of Alberta, Canada). The pET3a plasmids encoding human cardiac TnI and TnT were graciously provided by Dr. James Potter (University of Miami, FL). TnC, TnI and TnT mutants were constructed from their respective pET3a plasmids using the primer-based QuikChange Site-Directed Mutagenesis Kit (Stratagene, Santa Clara, CA) as previously described [11]. The mutations were confirmed by DNA sequence analysis at an on-site molecular genetics core facility.

### Protein Purification

The plasmid encoding human cardiac TnC was transformed into *E. coli* BL21(DE3)pLysS cells (Novagen, San Diego, CA), while the TnI and TnT plasmids were transformed into Rosetta™(DE3)pLysS cells (Novagen, San Diego, CA). The proteins were expressed and purified as previously described [25].

Rabbit skeletal actin and bovine ventricular tropomyosin (Tm) were purified from acetone powders as previously described [26], [27]. Fresh bovine cardiac muscle was purchased from The Herman Falter Packing Company (Columbus, OH). All the animal protocols and procedures were performed in accordance with the National Institutes of Health Guidelines and approved by the Institutional Laboratory Animal Care and Use Committee at The Ohio State University.

### Fluorescent Labeling

TnC^C35S,T53C,C84S^ (herein denoted as TnC^T53C^) was labeled with the environmentally sensitive thiol-reactive fluorescent probe IAANS as previously described [25].

### Reconstitution of Troponin Complexes and Regulated Thin Filaments

The reconstituted Tn complexes and regulated thin filaments were prepared as previously described [25]. All the Tn complexes contain full length Tn subunits of recombinant human 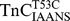, TnI (except for the truncated TnI (1-192)) and TnT.

### Steady-State Fluorescence Measurements

All steady-state fluorescence measurements were performed using a Perkin-Elmer LS55 spectrofluorimeter at 15°C. IAANS fluorescence was excited at 330 nm and monitored at 450 nm as microliter amounts of CaCl_2_ were added to 2 ml of each labeled Tn complex (0.15 µM) in a titration buffer (200 mM MOPS (to prevent pH changes upon addition of Ca^2+^), 150 mM KCl, 2 mM EGTA, 1 mM DTT, 3 mM MgCl_2_, 0.02% Tween-20, pH 7.0) with constant stirring. Reconstituted thin filaments were titrated in an identical buffer composition (excluding Tween-20). The [Ca^2+^]_free_ was calculated using the computer program EGCA02 developed by Robertson and Potter [28]. The Ca^2+^ sensitivities were reported as a dissociation constant K_d_, representing a mean of three to four separate titrations ± S.E.M. The data were fit with a logistic sigmoid function (mathematically equivalent to the Hill equation), as previously described [29].

### Stopped-Flow Fluorescent Measurements

Ca^2+^ exchange rates were characterized using an Applied Photophysics model SX.20 stopped-flow instrument with a dead time of 1.4 ms at 15°C. IAANS fluorescence was excited at 330 nm. The IAANS emission was monitored through either a 420-470 nm band-pass interference filter for 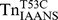, or a 510 nm broad band-pass interference filter for the thin filament. The filters were purchased from Oriel (Stratford, CT). Data traces (an average of 3 to 5 individual traces) were fit with a single exponential equation to calculate the kinetic rates. The working buffer used for the kinetic measurements was 10 mM MOPS, 150 mM KCl, 1 mM DTT, 3 mM MgCl_2_, 0.02% Tween-20 (excluded for thin filament kinetic measurements), at pH 7.0. 10 mM EGTA was utilized to remove 200 µM Ca^2+^ from the Tn complexes or thin filaments.

In order to measure the rate of Ca^2+^ association to the thin filament, a minimal concentration of EGTA was added to the thin filament mixture to remove contaminating Ca^2+^. The amount of EGTA added was determined by steady state titrations. For the control, TnI (1-192), TnT ΔK210, TnT R131W thin filaments, 5 µM EGTA was sufficient. For TnI R192H and TnI D190H thin filaments, 10 µM EGTA was required. The observed rates at various Ca^2+^ concentrations were plotted against the Ca^2+^ concentration and fit with a linear regression. The rate of Ca^2+^ association to the thin filament was obtained by calculating the slope of the fit [30]. The buffer used for the Ca^2+^ association rate measurements was the same as that used for the Ca^2+^ dissociation rate measurements.

### Exposure of the Thin Filament to Artificial Ca^2+^ Transients (ACTs)

In order to determine the potential effects of altering the Ca^2+^ association rate to the thin filament, the IAANS labeled TnCs on the thin filament were exposed to artificial Ca^2+^ transients (ACTs) of increasing amplitude as previously described [19], [31]. ACTs were generated in the stopped-flow apparatus by rapidly mixing the thin filament (at a concentration and buffer described above for the rates of Ca^2+^ dissociation) in the presence of 500 µM EGTA (after mixing) against increasing [Ca^2+^]. This technique exposes the thin filament to rapid transient fluxes of Ca^2+^ (∼ 1 ms half life). A relatively high concentration of EGTA (500 µM after mixing) was utilized to ensure that the thin filament is not pre-bound by Ca^2+^ and to provide a large enough Ca^2+^ sink to effectively remove the free [Ca^2+^] after mixing, effectively generating a Ca^2+^ transient. The amplitude of the Ca^2+^ transient can be adjusted by rapidly mixing the thin filament with different amounts of Ca^2+^. The ability of a Ca^2+^-binding protein (in this case, TnC on the thin filament) to respond to these rapid Ca^2+^ transients, or become transiently occupied by Ca^2+^, is a function of the Ca^2+^ association rate of the Ca^2+^-binding protein [31]. In order to determine the maximal response of the thin filaments to Ca^2+^, high [Ca^2+^] (1 mM after mixing) was used to exceed the [EGTA] and saturate the thin filament. Analysis of the response of the thin filament to the ACTs is described in the legend of Figure 7.

### Data Analysis and Statistics

Statistical significance was determined by ANOVA followed by a Dunnett’s post-hoc t-test using the statistical analysis software Minitab (State College, PA). Two means were considered to be significantly different when the P value was < 0.05. The data is shown as a mean value ± S.E.M.

## Results

### Effects of the Protein Modifications on the Ca^2+^ Sensitivity of the Tn Complex

The data were divided into four subgroups according to the disease subtypes to facilitate data presentation. The effects of the disease-related protein modifications on the Ca^2+^ binding properties of the Tn complex were examined since Tn is the simplest biochemical system to test the effects of TnI and TnT modifications on TnC. Our laboratory has developed a fluorescent TnC, 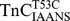 (which is specifically labeled with the fluorescent probe IAANS on Cys 53) that minimally affects TnC function, and reports the structural changes that occur within the regulatory domain of TnC upon Ca^2+^ binding and dissociation [11]. 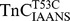 was incorporated into all the Tn complexes (denoted as 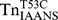), which enabled us to follow Ca^2+^ binding and exchange with isolated or thin filament bound Tn complexes.

**Figure 1:**
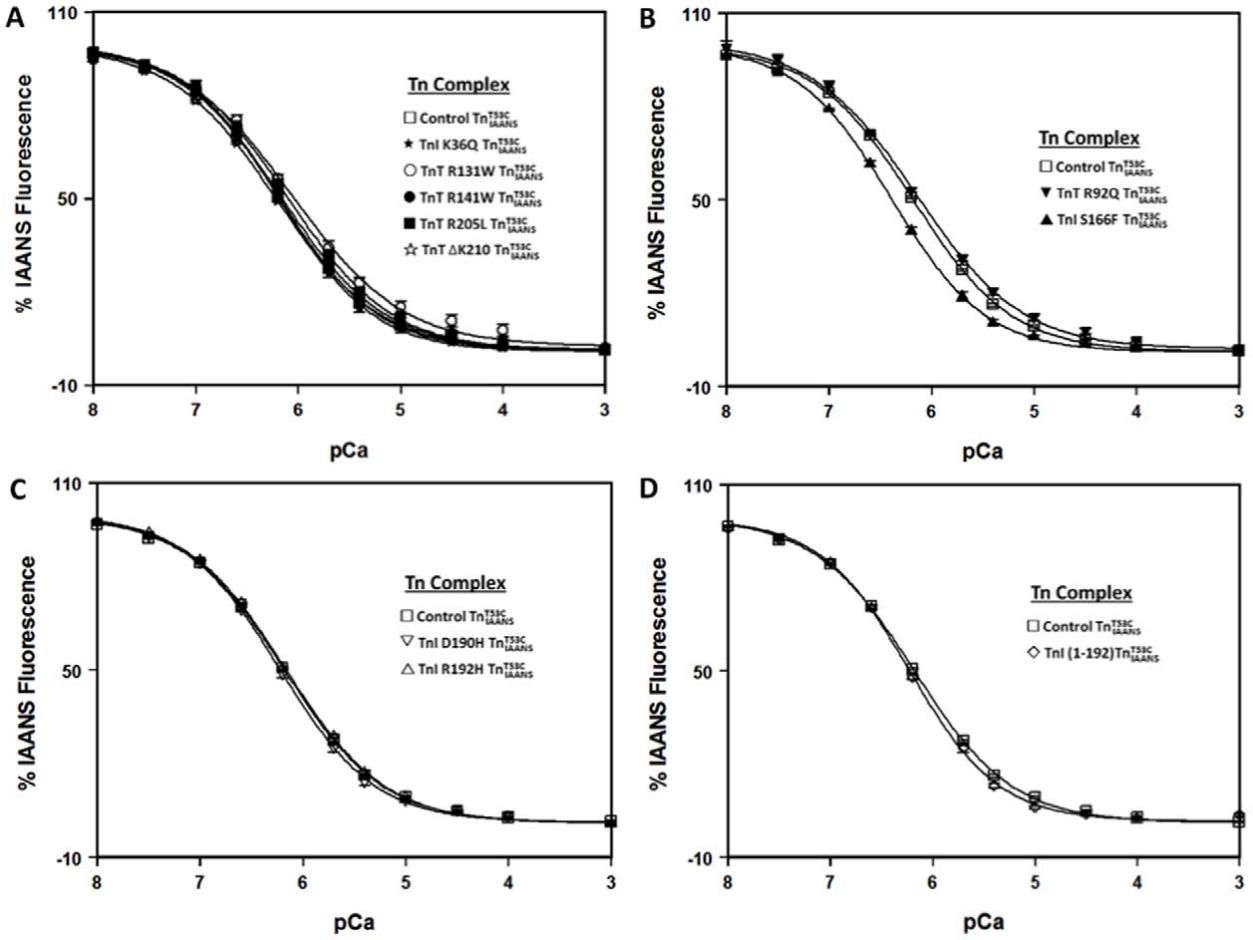
Effect of disease-related protein modifications on the Ca^2+^ sensitivity of the Tn complex. Panel A shows the Ca^2+^ dependent decreases in IAANS fluorescence for control 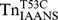 (□), and the DCM mutants, TnI K36Q 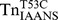 (?), TnT R131W 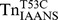 (○), TnT R141W 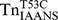 (•), TnT R205L 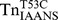 (▪) and TnT ΔK210 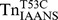 (⋆) as a function of pCa. Panel B shows the Ca^2+^ dependent decreases in IAANS fluorescence for control 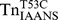 (□), and the HCM mutants, TnT R92Q 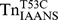 (▾) and TnI S166F 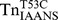 (▴) as a function of pCa. Panel C shows the Ca^2+^ dependent decreases in IAANS fluorescence for control 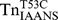 (□), and the RCM mutants, TnI D190H 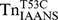 (▽) and TnI R192H 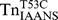 (▵) as a function of pCa. Panel D shows the Ca^2+^ dependent decreases in IAANS fluorescence for control 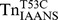 (□) and ischemic related truncated TnI (1-192) 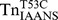 (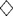) as a function of pCa. The data sets were normalized individually for each mutant. All 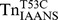 complexes consist of the full length Tn subunits of 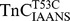, TnI and TnT, except for ischemic related truncated TnI (1-192). The disease related modification is either in TnI or TnT, in either case, the other protein (TnT or TnI) was wild type. The Ca^2+^ sensitivities were reported as a dissociation constant K_d_, representing a mean of three to four separate titrations ± S.E.M.

The Ca^2+^ sensitivity of TnC within the various Tn complexes was measured by following the Ca^2+^ dependent decrease in IAANS fluorescence. Control 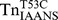 exhibited a Ca^2+^ induced half-maximal fluorescence decrease at 0.66±0.03 µM (Figure 1 and Table 1). As shown in figure 1, only HCM TnI S166F significantly increased the Ca^2+^ sensitivity of the Tn complex (∼ 1.5-fold; Figure 1 and Table 1).

**Table 1:** Effect of disease-related protein modifications on the Ca^2+^ binding properties of the Tn complex.

Protein	Disease Subtype	Tn complex Ca^2+^ K_d_ (µM)	Tn complex n_H_	Tn complex Ca^2+^ k_off_ (/s)
Control	–	0.66±0.03	0.90±0.02	40.8±0.4
TnI K36Q	DCM	0.68±0.07	0.95±0.05	48.5±0.3*
TnT R141W	DCM	0.71±0.09	0.87±0.04	39.3±0.3
TnT R131W	DCM	0.9±0.2	0.80±0.04	42.0±0.6
TnT R205L	DCM	0.80±0.06	0.86±0.06	41.6±0.5
TnT ΔK210	DCM	0.61±0.01	0.86±0.01	40.7±0.5
TnT R92Q	HCM	0.73±0.06	0.85±0.05	40±2
TnI S166F	HCM	0.43±0.04*	0.96±0.04	22.5±0.1*
TnI D190H	RCM	0.62±0.05	0.95±0.04	40.3±0.4
TnI R192H	RCM	0.674±0.007	0.89±0.03	41.2±0.6
TnI (1-192)	Ischemia injury	0.59±0.03	1.00±0.07	42.0±0.8

Values marked with * are significantly different from their respective control values (p<0.05).

### Effects of the Protein Modifications on the Rate of Ca^2+^ Dissociation from the Tn Complex

**Figure 2:**
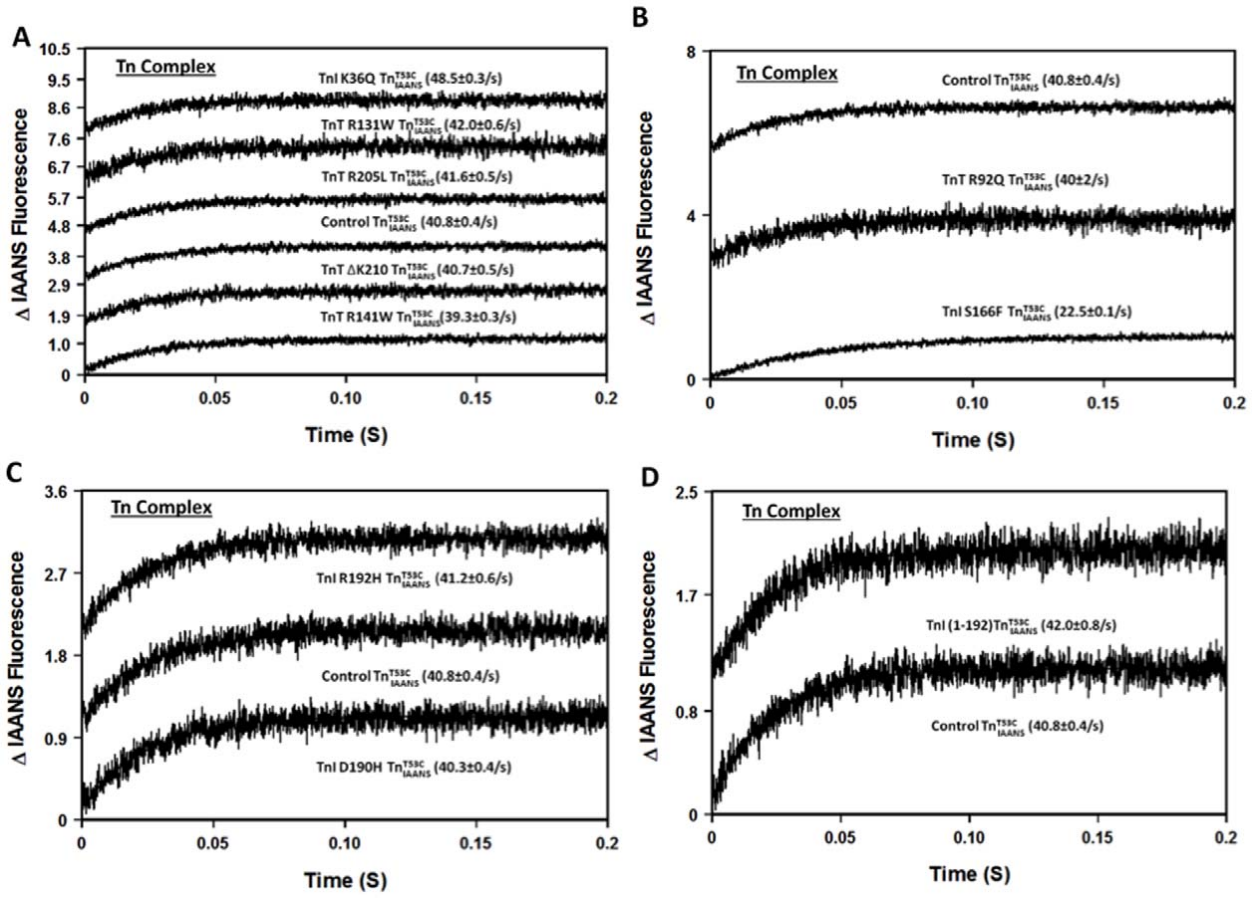
Effect of disease-related protein modifications on the rate of Ca^2+^ dissociation from the Tn complex. Panel A shows the time courses of the increase in IAANS fluorescence as Ca^2+^ was removed by EGTA from control 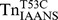, and the DCM mutants, TnI K36Q 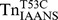, TnT R131W 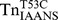, TnT R141W 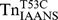, TnT R205L 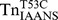 and TnT ΔK210 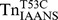. Panel B shows the time courses of the increase in IAANS fluorescence as Ca^2+^ was removed by EGTA from control 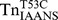, and the HCM mutants, TnT R92Q 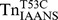 and TnI S166F 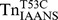. Panel C shows the time courses of the increase in IAANS fluorescence as Ca^2+^ was removed by EGTA from control 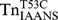, and the RCM mutants, TnI D190H 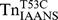 and TnI R192H 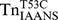. Panel D shows the time courses of the increase in IAANS fluorescence as Ca^2+^ was removed by EGTA from control 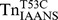 and ischemic related truncated TnI (1-192) 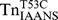. All 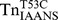 complexes consist of the full length Tn subunits of 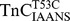, TnI and TnT, except for ischemic related truncated TnI (1-192). The disease related modification is either in TnI or TnT, in either case, the other protein (TnT or TnI) was wild type. Data traces (an average of 3 to 5 individual traces collected at least 10 times) were fit with a single exponential equation to calculate the kinetic rates. The data traces have been staggered and normalized for clarity.

Previously, we demonstrated that the fluorescence of 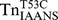 reported the actual rate of Ca^2+^ dissociation from unlabeled wild type Tn and a series of rationally engineered mutant Tn complexes with high fidelity [11], [25]. In this study, fluorescence stopped-flow measurements were performed to determine the rate of Ca^2+^ dissociation from the disease-related 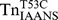 complexes. Figure 2 shows that the rate of Ca^2+^ dissociation from control 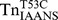 was 40.8±0.4/s (Table 1). Consistent with its effect on the Ca^2+^ sensitivity of Tn, HCM TnI S166F slowed the rate of Ca^2+^ dissociation ∼2-fold (Figure 2 and Table 1). Besides TnI S166F, only TnI K36Q slightly, but significantly altered the rate of Ca^2+^ dissociation from the Tn complex (∼ 1.2-fold faster; Figure 2A and Table 1).

### Effects of the Protein Modifications on the Ca^2+^ Sensitivity of the Thin Filament

Consistent with previous studies [6], [12], [13], our results indicate that the majority of the disease-related protein modifications have no effect on the Ca^2+^ binding properties of Tn. However, Tn is part of the thin filament system. Accordingly, we examined the effects of TnI and TnT modifications on the Ca^2+^ binding properties of the reconstituted thin filament.

**Figure 3:**
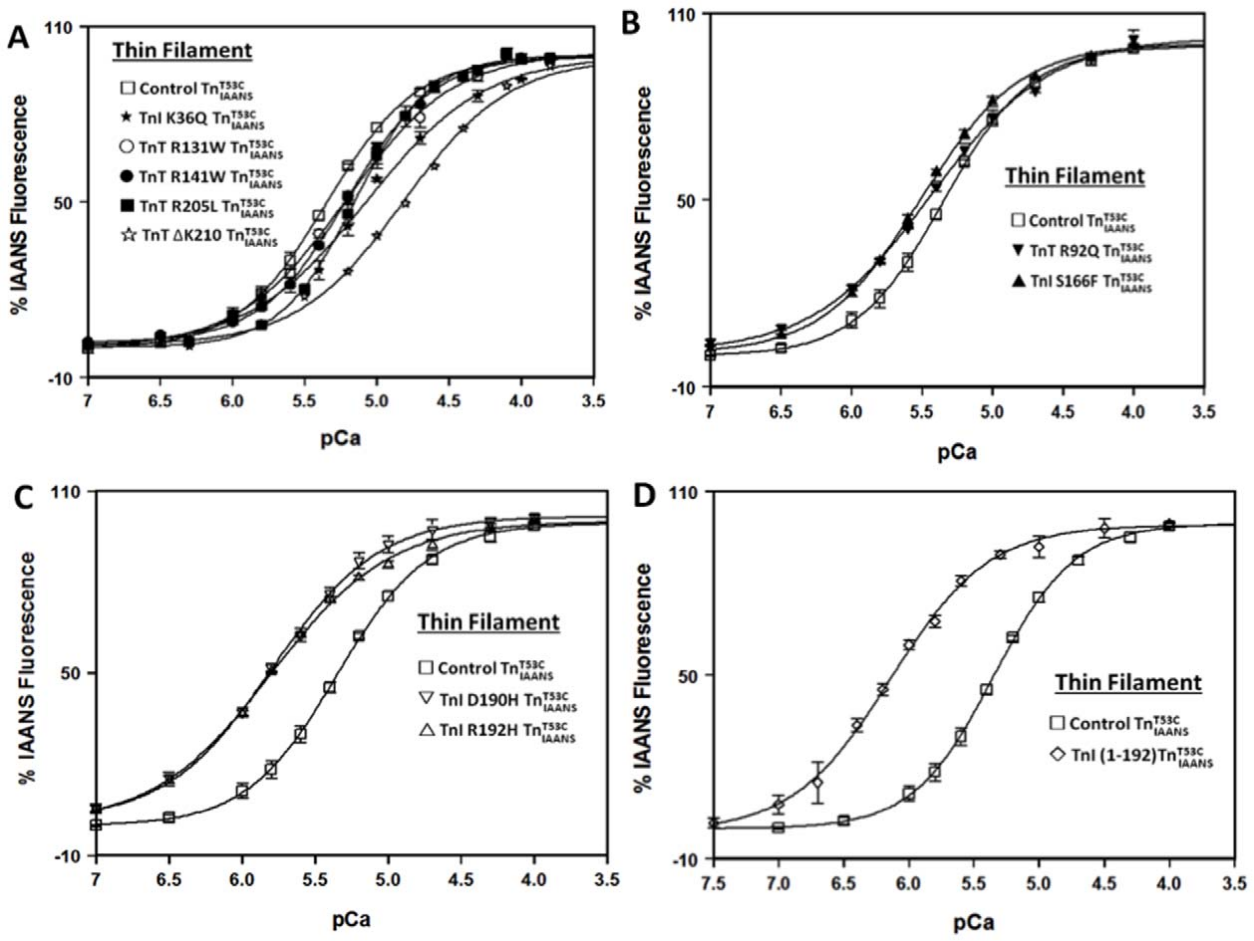
Effect of disease-related protein modifications on the Ca^2+^ binding sensitivity of the thin filament. Panel A shows the Ca^2+^ dependent increases in IAANS fluorescence for thin filament bound control 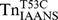 (□), and the DCM mutants, TnI K36Q 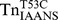 (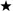), TnT R131W (○)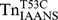, TnT R141W 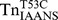 (•), TnT R205L 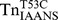 (▪) and TnT ΔK210 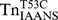 (⋆) as a function of pCa. Panel B shows the Ca^2+^ dependent increases in IAANS fluorescence for thin filament bound control 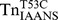 (□), and the HCM mutants, TnT R92Q 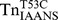 (▾) and TnI S166F 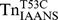 (▴) as a function of pCa. Panel C shows the Ca^2+^ dependent increases in IAANS fluorescence for thin filament bound control 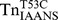 (□), and the RCM mutants, TnI D190H 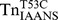 (▽) and TnI R192H 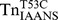 (▵) as a function of pCa. Panel D shows the Ca^2+^ dependent increases in IAANS fluorescence for thin filament bound control 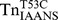 (□) and ischemic related truncated TnI (1-192) 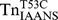 (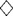) as a function of pCa. The data sets were normalized individually for each mutant. All 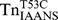 complexes consist of the full length Tn subunits of 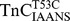, TnI and TnT, except for ischemic related truncated TnI (1-192). The disease related modification is either in TnI or TnT, in either case, the other protein (TnT or TnI) was wild type. The Ca^2+^ sensitivities were reported as a dissociation constant K_d_, representing a mean of three to four separate titrations ± S.E.M.

Thin filament bound control 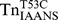 exhibited a Ca^2+^-dependent half-maximal fluorescence increase at 4.5±0.2 µM (Figure 3 and Table 2). Consistent with the general Ca^2+^ desensitizing effects of DCM mutations [5], [13], all five DCM mutations studied here desensitized Ca^2+^ binding to the thin filament. TnT R131W, R141W and R205L slightly desensitized Ca^2+^ binding to the thin filament by ∼ 1.5 fold; TnI K36Q had an intermediate Ca^2+^ desensitizing effect of ∼ 2-fold; while TnT ΔK210 had the largest Ca^2+^ desensitizing effect of ∼ 3.5-fold (Figure 3C and Table 2). Of the Ca^2+^ desensitizing modifications, only TnT ΔK210 significantly decreased the Ca^2+^ binding cooperativity of the thin filament by ∼ 1.3-fold (Figure 3C and Table 2).

**Table 2:** Effect of disease-related protein modifications on the Ca^2+^ binding properties of the thin filament.

Protein	Disease Subtype	TF Ca^2+^ K_d_ (µM)	Relative Changein K_d_	TF n_H_	TF Ca^2+^ k_off_ (/s)	Relative Change in k_off_	Calculated TF Ca^2+^ k_on_ (×10^6^ M-^1^s-^1^)	TF Ca^2+^ k_on_ (×10^6^ M^-1^s^-1^)
Control	–	4.5±0.2	–	1.4±0.1	109.7±0.7	–	24±1	9±1
TnI K36Q	DCM	8.4±0.3*	↓1.9±0.1	1.1±0.1	279±5*	↑2.54±0.05	33±1	ND
TnT R141W	DCM	6.2±0.1*	↓1.38±0.07	1.44±0.04	115.0±0.8	1.05±0.01	18.5±0.3	ND
TnT R131W	DCM	6.2±0.4*	↓1.4±0.1	1.16±0.04	138±1*	↑1.26±0.01	22±1	12±1
TnT R205L	DCM	7.2±0.6*	↓1.6±0.2	1.7±0.1	166±2*	↑1.51±0.02	23±2	ND
TnT ΔK210	DCM	15±1*	↓3.3±0.3	1.05±0.06*	252±6*	↑2.30±0.06	17±1	3.6±0.6
TnT R92Q	HCM	3.7±0.1	↑1.22±0.06	1.05±0.04*	107.5±0.8	1.02±0.01	29.1±0.8	ND
TnI S166F	HCM	3.1±0.1*	↑1.45±0.08	1.24±0.04	65.2±0.4*	↓1.68±0.01	21.0±0.7	ND
TnI D190H	RCM	1.62±0.03*	↑2.8±0.1	1.2±0.1	87.9±0.6*	↓1.24±0.01	54±1	36±3
TnI R192H	RCM	1.59±0.01*	↑2.8±0.1	1.17±0.05	72±1*	↓1.52±0.02	45.3±0.7	48±5
TnI (1-192)	Ischemia injury	0.71±0.05*	↑6.3±0.5	1.21±0.06	55.3±0.4*	↓1.98±0.02	78±6	49±11

Values marked with * are significantly different from their respective control values (p<0.05). ND: not determined.

Consistent with the general Ca^2+^ sensitizing effect of HCM, RCM and ischemia-induced truncation of TnI [5], [6], four of the five TnI and TnT modifications studied here sensitized Ca^2+^ binding to the thin filament. As shown in figure 3B, HCM TnI S166F slightly, but significantly sensitized Ca^2+^ binding to the thin filament by ∼ 1.4-fold. However, the other HCM mutation R92Q did not significantly alter thin filament Ca^2+^ sensitivity, but significantly decreased the Ca^2+^ binding cooperativity of the thin filament by ∼ 1.3-fold (Figure 3B and Table 2).

Figure 3C shows that both RCM mutations, TnI D190H and TnI R192H, increased the Ca^2+^ sensitivity of the thin filament by ∼ 2.8-fold (Figure 3C and Table 2). Compared to the HCM and RCM mutations, which had slight and intermediate Ca^2+^ sensitizing effects, the ischemia-induced truncation of TnI (1-192) hyper-sensitized Ca^2+^ binding to thin filament by ∼ 6.3-fold (Figure 3D and Table 2).

### Effect of the Protein Modifications on the Rate of Ca^2+^ Dissociation from the Thin Filament

**Figure 4:**
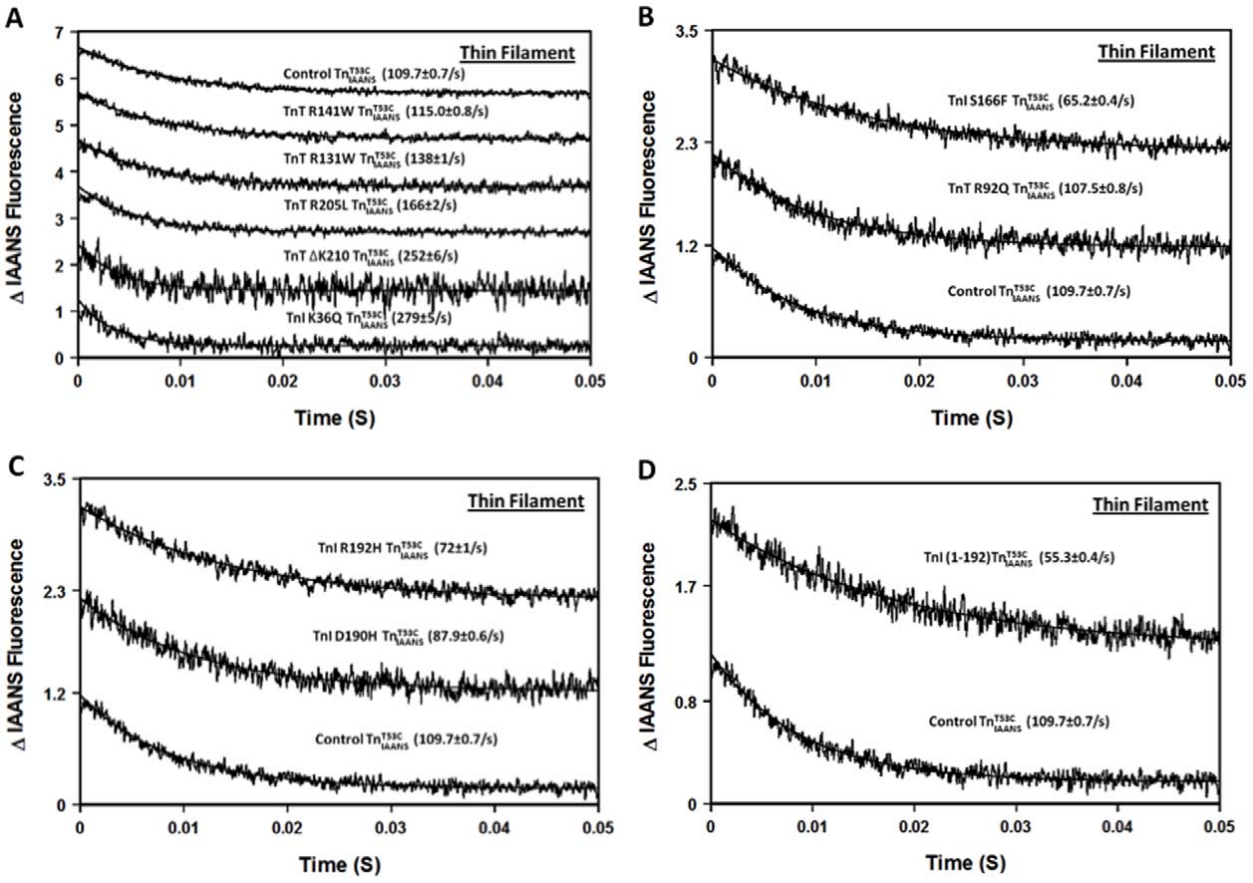
Effect of disease-related protein modifications on the rate of Ca^2+^ dissociation from the thin filament. Panel A shows the time courses of the decrease in IAANS fluorescence as Ca^2+^ was removed by EGTA from thin filament bound control 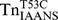, and the DCM mutants, TnI K36Q 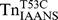, TnT R131W 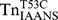, TnT R141W 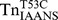, TnT R205L 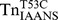 and TnT ΔK210 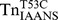. Panel B shows the time courses of the decrease in IAANS fluorescence as Ca^2+^ was removed by EGTA from thin filament bound control 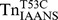, and the HCM mutants, TnT R92Q 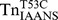 and TnI S166F 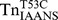. Panel C shows the time courses of the decrease in IAANS fluorescence as Ca^2+^ was removed by EGTA from thin filament bound control 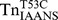, and the RCM mutants, TnI D190H 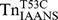 and TnI R192H 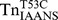. Panel D shows the time courses of the decrease in IAANS fluorescence as Ca^2+^ was removed by EGTA from thin filament bound control 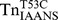 and ischemic related truncated TnI (1-192) 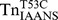. All 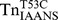 complexes consist of the full length Tn subunits of 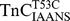, TnI and TnT, except for ischemic related truncated TnI (1-192). The disease related modification is either in TnI or TnT, in either case, the other protein (TnT or TnI) was wild type. Data traces (an average of 3 to 5 individual traces collected at least 10 times) were fit with a single exponential equation to calculate the kinetic rates. The data traces have been staggered and normalized for clarity.

It is generally assumed that a change in the Ca^2+^ sensitivity of TnC is due to a change in the rate of Ca^2+^ dissociation. Figure 4A shows that the rate of Ca^2+^ dissociation from thin filament bound control 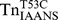 was 109.7±0.7/s (Table 2). Figure 4A also shows the effects of the five DCM mutations on the rate of Ca^2+^ dissociation from the thin filament. TnT R141W negligibly altered the rate of Ca^2+^ dissociation, while TnT R131W and R205L caused a slightly faster rate of Ca^2+^ dissociation from the thin filament (less than ∼ 1.5-fold; Figure 4A and Table 2). TnI K36Q and TnT ΔK210 had larger effects on accelerating the rate of Ca^2+^ dissociation from the thin filament (∼ 2.5-fold and ∼ 2.3-fold, respectively; Table 2).

Figure 4B, C and D show the effects of the two HCM mutations, two RCM mutations and ischemia-induced truncation of TnI on the rate of Ca^2+^ dissociation from the thin filament. The HCM mutation TnI S166F slowed the rate of Ca^2+^ dissociation by ∼ 1.7-fold, while the HCM mutation TnT R92Q had no effect on the dissociation rate (Figure 4B and Table 2). Both RCM mutations slowed the rate of Ca^2+^ dissociation from the thin filament, ∼1.2-fold slower for TnI D190H and ∼1.5-fold slower for TnI R192H (Figure 4C and Table 2). The ischemia-induced truncation of TnI (1-192) slowed the rate of Ca^2+^ dissociation from the thin filament by ∼ 2-fold (Figure 4D and Table 2).

### Effects of the Protein Modifications on the Rate of Ca^2+^ Association to the Thin Filament

**Figure 5:**
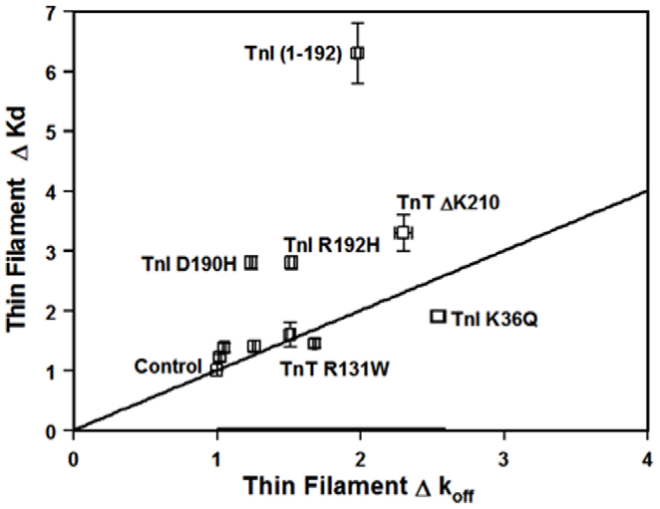
The relationship between changes in the Ca^2+^ sensitivity and the rate of Ca^2+^ dissociation. The changes in the thin filament Ca^2+^ sensitivity for the ten disease-related protein modifications are plotted against the changes in the rate of Ca^2+^ dissociation from the thin filament. The straight line in the figure represents a perfect correlation between the thin filament change in Ca^2+^ sensitivity and Ca^2+^ dissociation rate.

**Figure 6:**
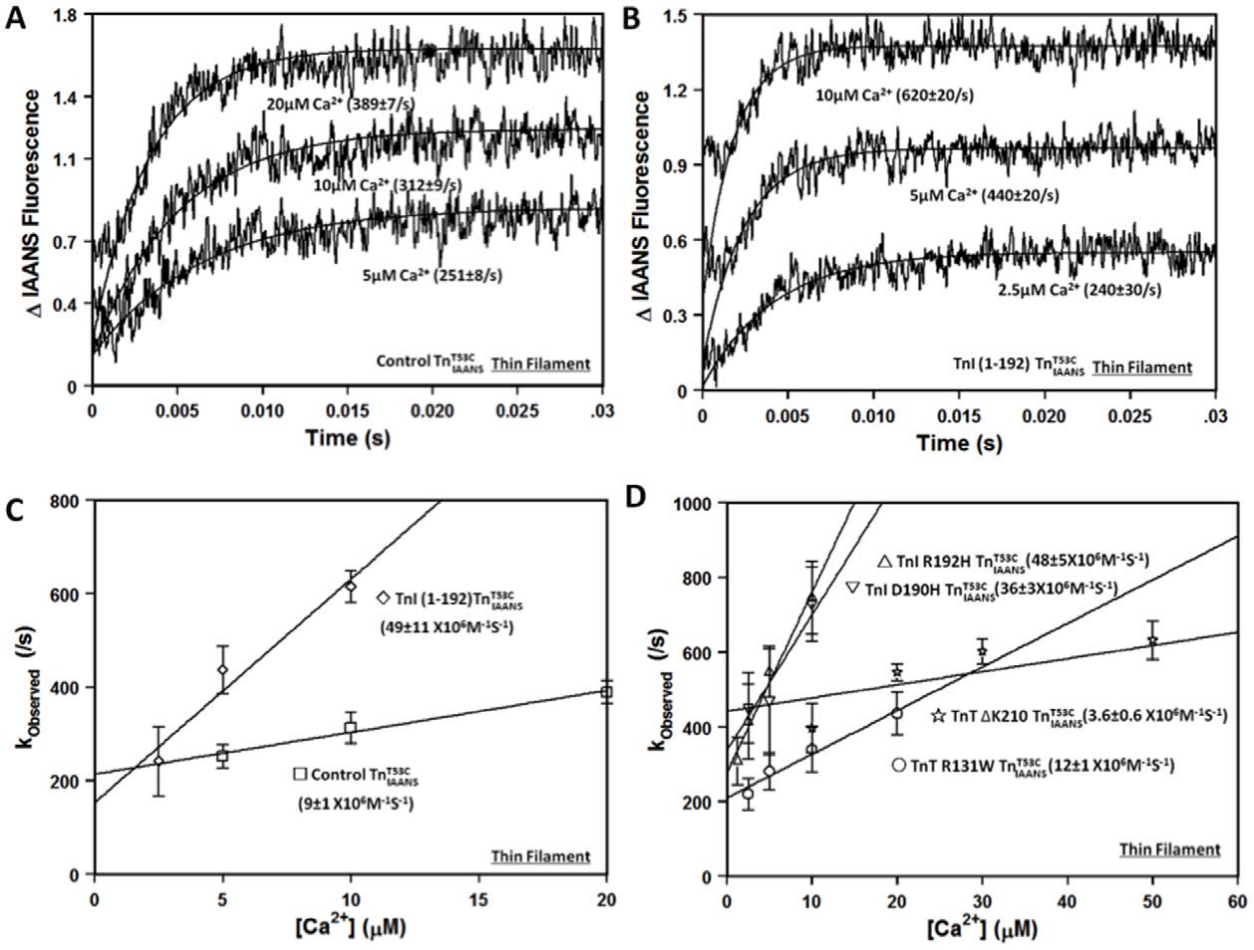
Effect of disease-related protein modifications on the rate of Ca^2+^ association to the thin filament. Panel A shows the kinetic traces (an average of 3 to 5 individual traces collected at least 9 times) observed for Ca^2+^ association to thin filament bound control 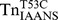 at 5 µM, 10 µM and 20 µM Ca^2+^ ([Ca^2+^] after mixing). Panel B shows the kinetic traces (an average of 3 to 5 individual traces collected at least 9 times) observed for Ca^2+^ association to thin filament bound TnI(1-192) 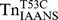 at 2.5 µM, 5 µM and 10 µM Ca^2+^. The rate of the fluorescence increase when Ca^2+^ associates with the thin filament was fit with a single exponential to calculate the observed rate (k_observed_). All 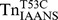 complexes consist of the full length Tn subunits of 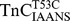, TnI and TnT, except for ischemic related truncated TnI (1-192). The disease related modification is either in TnI or TnT, in either case, the other protein (TnT or TnI) was wild type. Panel C shows the plots of k_observed_ versus [Ca^2+^] for thin filament bound control 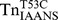 (□) and TnI (1-192) 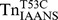 (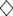). The rate of Ca^2+^ association to the thin filament was obtained by calculating the slope of a linear fit to the data. Panel D shows the plots of k_observed_ versus [Ca^2+^] for thin filament bound TnI R192H 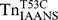 (▵), TnI D190H 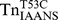 (▽), TnT ΔK210 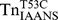 (⋆), and TnT R131W 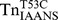 (○).

The kinetic studies indicate that the changes in Ca^2+^ sensitivity of the thin filament caused by the protein modifications are in part due to changes in the rate of Ca^2+^ dissociation. However, as shown in table 2 and figure 5, there was only a weak correlation (r^2^ = 0.17, fit not shown) between the changes in the thin filament Ca^2+^ sensitivities and the changes in the Ca^2+^ dissociation rates for the different protein modifications. These results suggest that the rate of Ca^2+^ association to the thin filament can also be altered by the protein modifications. To test this hypothesis, stopped-flow experiments were performed to measure the rate of Ca^2+^ association to the thin filaments containing TnI R192H, D190H, TnI (1-192), TnT ΔK210 and TnT R131W. Based on the calculated rate of Ca^2+^ association, TnT R131W was predicted to have little effect on the rate of Ca^2+^ association while the rest of the protein modifications were expected to significantly change the rate of Ca^2+^ association (Table 2). Using a traditional approach to determine the apparent Ca^2+^ association rate to the thin filament, the measured rate of Ca^2+^ association to the control thin filament was 9±1×10^6^ M^−1^s^−1^ (Figure 6A, 6C and Table 2). Consistent with the calculated Ca^2+^ association rates, TnI R192H, D190H and TnI (1-192) increased the apparent rate of Ca^2+^ association to the thin filament by ∼ 4- to 5-fold (Figure 6B, 6D and Table 2). In contrast, TnT ΔK210 slowed the apparent rate of Ca^2+^ association to the thin filament by ∼3-fold (Figure 6D and Table 2). On the other hand, the apparent rate of Ca^2+^ association to the thin filament containing TnT R131W was similar to that of the control thin filament, with the rate being 12±1×10^6^ M^−1^s^−1^ (Figure 6D and Table 2). Thus, the experimental results indicate that the apparent rate of Ca^2+^ association to the thin filament can also be altered by the disease-related protein modifications.

**Figure 7:**
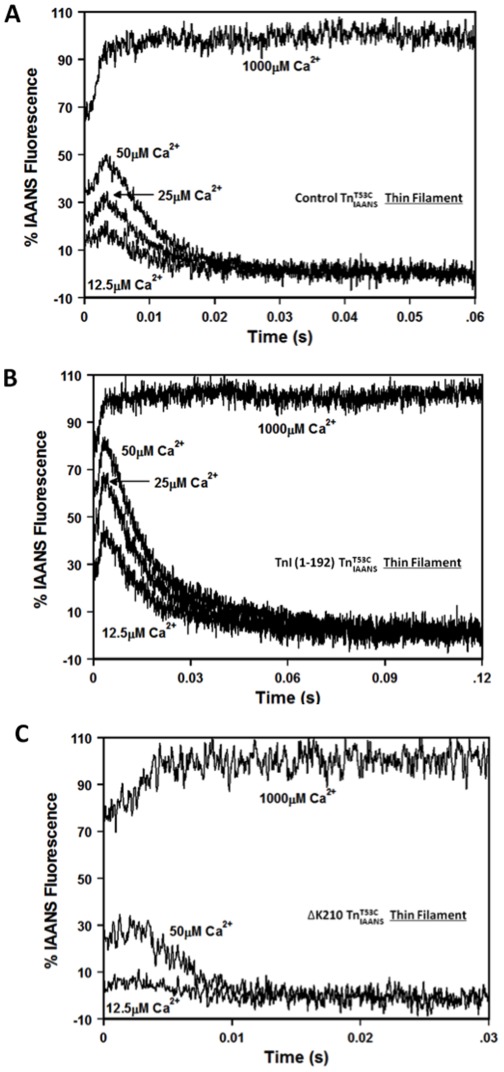
Response of disease-related protein modifications to ACTs with increasing amplitude. Panels A, B and C show responses of thin filament bound control 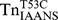, TnI (1-192) 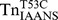 and TnT ▵K210 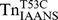 to ACTs, respectively. The peak transient occupancy was determined at ∼3 ms for each sub-saturating [Ca^2+^]. 100% occupancy was determined at the plateau of the trace in which saturating Ca^2+^ (1000 µM after mixing) was rapidly mixed with the thin filament. 0% occupancy was determined by mixing the thin filament without Ca^2+^ (data not shown). Each transient occupancy calculation was an average of three separate experiments repeated twice, with each trace being an average of at least 5 separate traces. The 25 µM Ca^2+^ data for TnT ▵K210 is not shown for clarity. All 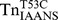 complexes consist of the full length Tn subunits of 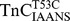, TnI and TnT, except for ischemic related truncated TnI (1-192). The disease related modification is either in TnI or TnT, in either case, the other protein (TnT or TnI) was wild type.

The Ca^2+^ association rate data suggest that the disease-related modifications alter the ability of the thin filament to respond to a Ca^2+^ transient. This idea was tested in a stopped-flow apparatus by exposing the thin filament to artificial Ca^2+^ transients (ACTs) of increasing amplitude (Figure 7). Due to the relatively slow Ca^2+^ association rate of EGTA, initially Ca^2+^ transiently binds to the thin filament (dependent on its Ca^2+^ association rate) until EGTA chelates the Ca^2+^ (∼ 1 ms half life). Although these are not physiological Ca^2+^ transients, it is expected that for a fixed Ca^2+^ transient amplitude, a faster rate of Ca^2+^ association should lead to a higher level of transient occupancy, while a slower rate of Ca^2+^ association should lead to a lower level of transient occupancy. Figure 7A shows the responses of thin filament bound control 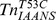 to ACTs. As the [Ca^2+^] was successively increased from 12.5 µM to 25 µM and then to 50 µM (after mixing), the percentage of transient occupancy increased from 17±1%, to 30±2% and then to 47±3%, respectively (Table 3). Compared to the control thin filament, the percentage of the transient occupancy was higher for TnI (1-192) and lower for TnT ΔK210 at all three Ca^2+^ concentrations (Figure 7B, 7C and Table 3). Thus, consistent with the Ca^2+^ association rate calculations and measurements, the ACT experiments support the idea that the TnI (1-192) containing thin filament has an apparent faster rate of Ca^2+^ association, while the TnT ΔK210 containing thin filament has an apparent slower rate of Ca^2+^ association than the control thin filament.

**Table 3:** Percent transient occupancy of the thin filament during artificial Ca^2+^ transients of increasing amplitude.

Protein	% Transient Occupancywith 12.5 µM Ca^2+^	% Transient Occupancywith 25 µM Ca^2+^	% Transient Occupancywith 50 µM Ca^2+^
Control	17±1	30±2	47±3
TnT ▵K210	5±3^*^	20±2^*^	28±6^*^
TnI (1–192)	44±3^*^	64±2^*^	81±2^*^

Values marked with * are significantly different from their respective control values (p<0.05).

## Discussion

In this study, we examined the effects of cardiac disease-related TnI and TnT modifications on the Ca^2+^ binding properties of TnC in the Tn complex and on the thin filament. The selected ten protein modifications are associated with a broad range of cardiac diseases: three subtypes of familial cardiomyopathies (DCM, HCM and RCM), and ischemia-reperfusion injury. Most of the selected protein modifications have previously been shown to alter the Ca^2+^ sensitivity of skinned cardiac muscle force generation, and/or the actomyosin-ATPase activity [12], [13], [21], [22]. Transgenic mice models have been developed for TnI R192H, TnI (1-192), TnT R92Q, TnT ΔK210 and TnT R141W, all of which recapitulate the phenotypes of the human diseases [23], [24], [32], [33], [34]. In addition to altering the steady-state Ca^2+^ sensitivity of force generation, some of these protein modifications also alter the kinetics and magnitude of contraction and/or relaxation [23], [24]. Thus, it is important to understand not only the effects of these protein modifications on the Ca^2+^ sensitivity of TnC, but also the effects on the kinetics of Ca^2+^ exchange with TnC.

Previous studies utilized IAANS labeled fluorescent TnCs (at either Cys 35 or Cys 84) to measure the effects of disease-related protein modifications on the steady-state Ca^2+^ sensitivity of TnC [6], [12], [13], [22]. Our steady-state results were qualitatively consistent with these previous studies. For instance, we observed that the majority of the disease-related protein modifications did not alter the Ca^2+^ binding properties of the Tn complex [6], [12], [13], [22]. However, when reconstituted into the thin filament, the DCM mutations decreased the Ca^2+^ sensitivity of the thin filament, while most of the HCM and RCM mutations, as well as the ischemia-induced truncation of TnI, increased the Ca^2+^ sensitivity of the thin filament [6], [12], [13], [22]. Slight quantitative differences in the Ca^2+^ sensitivity observed for the same protein modifications in the current study, compared to previous studies [6], [12], [13], [22], might be due to the utilization of different fluorescent TnCs, protein isoforms from different species (human in this manuscript), buffer composition, temperature, or the integrity of the Tn complex and/or the reconstituted thin filament. Great care was taken in the current study to ensure that there was no contaminating free TnC within the Tn complex, or free Tn within the thin filament that would have compromised the results.

The steady-state Ca^2+^ sensitivity of TnC is determined by the kinetics of Ca^2+^ association and dissociation. It is generally assumed that alterations in the steady-state Ca^2+^ sensitivity of TnC operate exclusively through changes in the rate of Ca^2+^ dissociation, since Ca^2+^ association to TnC has been traditionally thought to be diffusion controlled (for review see [15]). Our data clearly indicate that the majority of the disease-related protein modifications altered the rate of Ca^2+^ dissociation from the thin filament, in a way that is consistent with their effect on Ca^2+^ sensitivity. However, the magnitude of the change in the Ca^2+^ dissociation rates does not always correlate with the magnitude of the Ca^2+^ sensitivity changes. This finding suggests that the Ca^2+^ association rate to TnC must also be affected by the disease-related protein modifications. Experimental measurements verified that the apparent rates of Ca^2+^ association were also altered by some of the disease-related protein modifications. These data are consistent with our previous results that demonstrated natural and engineered TnC mutations could also affect the rate of Ca association to TnC [19], [20]. Interestingly, the Ca^2+^ sensitizing protein modifications (TnI R192H, D190H, and ischemia-induced truncation of TnI) tend to alter the Ca^2+^ binding sensitivity by modulating both the association and dissociation rates; while the Ca^2+^ desensitizing mutations tend to predominantly affect the dissociation rates (with the exception of TnT ΔK210).

While the physiological significance of the Ca^2+^ dissociation rate from TnC remains controversial [15], it is striking that Tn modifications (disease or engineered) with slowed or accelerated Ca^2+^ dissociation rates prolonged or abbreviated relaxation [23], [24], [35]. Thus, an aberrant rate of Ca^2+^ dissociation from TnC could potentially contribute to the diastolic dysfunction typically observed with the various cardiomyopathies [36], [37]. On the other hand, the rate of Ca^2+^ association to the thin filament could affect the amount of maximal force generation or myocyte shortening. For instance, despite similar intracellular Ca^2+^ transients, myocytes transfected with TnI R192H had a larger shortening amplitude than control myocytes, especially under high stimulating frequencies [38]. Whereas, intact papillary muscles from TnT ΔK210 transgenic mice displayed the same amount of maximal force as control muscles, even though the amplitude of the intracellular Ca^2+^ transient was higher than that of the control [24]. These transgenic studies suggest that in addition to the intracellular Ca^2+^ transient, the response of the thin filament to Ca^2+^ may also regulate the extent and duration of force development. Consistent with this idea, the current study demonstrates that both TnI (1-192) and TnT ΔK210 altered the response of the thin filament to non-physiological, artificial Ca^2+^ transients in a way consistent with their contractile effects.

There are potentially multiple mechanisms that exist to alter the Ca^2+^ binding properties of TnC on the thin filament [15]. It is well established that the binding of TnI to TnC is critical in determining the Ca^2+^ sensitivity and kinetics of TnC [11], [15]. However, it appears that only TnI S166F altered the ability of TnI to directly change the Ca^2+^ binding properties of TnC, since it was the only protein modification that substantially altered the Ca^2+^ binding properties of the Tn complex. TnI S166F is located close to the switch region of TnI that binds to the hydrophobic pocket of the regulatory domain of TnC. Thus, TnI S166F might be influencing this critical TnC-TnI interaction to affect the Ca^2+^ binding properties of TnC.

The more distant C-terminal region of TnI (residues 188-210) is believed to contribute to the inhibition of actomyosin interactions during diastole by direct interactions with actin-Tm [39]. The ability of TnI to bind actin-Tm may reduce the ability of the switch region of TnI to interact with the hydrophobic pocket of TnC, in essence reducing the apparent Ca^2+^ sensitivity of TnC [11]. TnI D190H, R192H and the removed residues from TnI (1-192) are located within this region of TnI and may weaken the interactions between TnI and actin-Tm. In this regard, these protein modifications may facilitate the switching of TnI from actin-Tm to TnC, causing an enhanced apparent sensitivity for Ca^2+^ only when the Tn complex is incorporated in the thin filament. Similarly, the TnT mutations may also change the ability of TnI to bind to actin-Tm. This hypothesis would be consistent with the TnT modifications only affecting the apparent Ca^2+^ sensitivity of the thin filament and not the Tn complex.

In summary, the disease-related protein modifications studied in this work adversely altered the steady-state Ca^2+^ sensitivity of the thin filament by influencing both the association and dissociation rates of Ca^2+^ exchange. The protein modifications modified the Ca^2+^ exchange properties of TnC in a way consistent with their effect on cardiac muscle physiology. Therefore, TnC may act as a central hub that converges these pathological stimuli to affect cardiac contractile properties. The abnormal myofilament Ca^2+^ binding along with aberrant intra-cellular Ca^2+^ signaling could contribute to contractile dysfunctions leading to cardiac remodeling and disease phenotypes. Thus, pharmaceutically [40] or genetically targeting TnC to correct the abnormal myofilament Ca^2+^ binding properties may represent a beneficial therapeutic strategy.

## References

[1] Janssen PM (2010). Myocardial contraction-relaxation coupling.. Am J Physiol Heart Circ Physiol.

[2] O’Rourke B, Kass DA, Tomaselli GF, Kaab S, Tunin R (1999). Mechanisms of altered excitation-contraction coupling in canine tachycardia-induced heart failure, I: experimental studies.. Circ Res.

[3] Bers DM, Despa S, Bossuyt J (2006). Regulation of Ca2+ and Na+ in normal and failing cardiac myocytes.. Ann N Y Acad Sci.

[4] van der Velden J (2011). Diastolic myofilament dysfunction in the failing human heart.. Pflugers Arch.

[5] Willott RH, Gomes AV, Chang AN, Parvatiyar MS, Pinto JR (2010). Mutations in Troponin that cause HCM, DCM AND RCM: what can we learn about thin filament function?. J Mol Cell Cardiol.

[6] Tachampa K, Kobayashi T, Wang H, Martin AF, Biesiadecki BJ (2008). Increased cross-bridge cycling kinetics after exchange of C-terminal truncated troponin I in skinned rat cardiac muscle.. J Biol Chem.

[7] Mirza M, Marston S, Willott R, Ashley C, Mogensen J (2005). Dilated cardiomyopathy mutations in three thin filament regulatory proteins result in a common functional phenotype.. J Biol Chem.

[8] Gomes AV, Liang J, Potter JD (2005). Mutations in human cardiac troponin I that are associated with restrictive cardiomyopathy affect basal ATPase activity and the calcium sensitivity of force development.. J Biol Chem.

[9] Tardiff JC (2011). Thin filament mutations: developing an integrative approach to a complex disorder.. Circ Res.

[10] Gordon AM, Homsher E, Regnier M (2000). Regulation of contraction in striated muscle.. Physiol Rev.

[11] Davis JP, Norman C, Kobayashi T, Solaro RJ, Swartz DR (2007). Effects of thin and thick filament proteins on calcium binding and exchange with cardiac troponin C. Biophys J.

[12] Kobayashi T, Solaro RJ (2006). Increased Ca2+ affinity of cardiac thin filaments reconstituted with cardiomyopathy-related mutant cardiac troponin I. J Biol Chem.

[13] Robinson P, Griffiths PJ, Watkins H, Redwood CS (2007). Dilated and hypertrophic cardiomyopathy mutations in troponin and alpha-tropomyosin have opposing effects on the calcium affinity of cardiac thin filaments.. Circ Res.

[14] Lu QW, Hinken AC, Patrick SE, Solaro RJ, Kobayashi T (2010). Phosphorylation of cardiac troponin I at protein kinase C site threonine 144 depresses cooperative activation of thin filaments.. J Biol Chem.

[15] Davis JP, Tikunova SB (2008). Ca(2+) exchange with troponin C and cardiac muscle dynamics.. Cardiovasc Res.

[16] Dong WJ, Xing J, Ouyang Y, An J, Cheung HC (2008). Structural kinetics of cardiac troponin C mutants linked to familial hypertrophic and dilated cardiomyopathy in troponin complexes.. J Biol Chem.

[17] Iorga B, Blaudeck N, Solzin J, Neulen A, Stehle I (2008). Lys184 deletion in troponin I impairs relaxation kinetics and induces hypercontractility in murine cardiac myofibrils.. Cardiovasc Res.

[18] Kruger M, Zittrich S, Redwood C, Blaudeck N, James J (2005). Effects of the mutation R145G in human cardiac troponin I on the kinetics of the contraction-relaxation cycle in isolated cardiac myofibrils.. J Physiol.

[19] Tikunova SB, Davis JP (2004). Designing calcium-sensitizing mutations in the regulatory domain of cardiac troponin C. J Biol Chem.

[20] Liang B, Chung F, Qu Y, Pavlov D, Gillis TE (2008). Familial hypertrophic cardiomyopathy-related cardiac troponin C mutation L29Q affects Ca2+ binding and myofilament contractility.. Physiol Genomics.

[21] Morimoto S, Lu QW, Harada K, Takahashi-Yanaga F, Minakami R (2002). Ca(2+)-desensitizing effect of a deletion mutation Delta K210 in cardiac troponin T that causes familial dilated cardiomyopathy.. Proc Natl Acad Sci U S A.

[22] Carballo S, Robinson P, Otway R, Fatkin D, Jongbloed JD (2009). Identification and functional characterization of cardiac troponin I as a novel disease gene in autosomal dominant dilated cardiomyopathy.. Circ Res.

[23] Du J, Liu J, Feng HZ, Hossain MM, Gobara N (2008). Impaired relaxation is the main manifestation in transgenic mice expressing a restrictive cardiomyopathy mutation, R193H, in cardiac TnI.. Am J Physiol Heart Circ Physiol.

[24] Du CK, Morimoto S, Nishii K, Minakami R, Ohta M (2007). Knock-in mouse model of dilated cardiomyopathy caused by troponin mutation.. Circ Res.

[25] Tikunova SB, Liu B, Swindle N, Little SC, Gomes AV (2010). Effect of calcium-sensitizing mutations on calcium binding and exchange with troponin C in increasingly complex biochemical systems.. Biochemistry.

[26] Smillie LB (1982). Preparation and identification of alpha- and beta-tropomyosins..

[27] Pardee JD, Spudich JA (1982). Purification of muscle actin..

[28] Robertson S, Potter JD (1984). The regulation of free Ca2+ ion concentration by metal chelators.. Methods in Pharmacology.

[29] Tikunova SB, Rall JA, Davis JP (2002). Effect of hydrophobic residue substitutions with glutamine on Ca(2+) binding and exchange with the N-domain of troponin C. Biochemistry.

[30] Kasturi R, Vasulka C, Johnson JD (1993). Ca2+, caldesmon, and myosin light chain kinase exchange with calmodulin.. J Biol Chem.

[31] Davis JP, Tikunova SB, Walsh MP, Johnson JD (1999). Characterizing the response of calcium signal transducers to generated calcium transients.. Biochemistry.

[32] Murphy AM, Kogler H, Marban E (2000). A mouse model of myocardial stunning.. Mol Med Today.

[33] Tardiff JC, Hewett TE, Palmer BM, Olsson C, Factor SM (1999). Cardiac troponin T mutations result in allele-specific phenotypes in a mouse model for hypertrophic cardiomyopathy.. J Clin Invest.

[34] Juan F, Wei D, Xiongzhi Q, Ran D, Chunmei M (2008). The changes of the cardiac structure and function in cTnTR141W transgenic mice.. Int J Cardiol.

[35] Kreutziger KL, Piroddi N, McMichael JT, Tesi C, Poggesi C (2011). Calcium binding kinetics of troponin C strongly modulate cooperative activation and tension kinetics in cardiac muscle.. J Mol Cell Cardiol.

[36] Maron BJ, Towbin JA, Thiene G, Antzelevitch C, Corrado D (2006). Contemporary definitions and classification of the cardiomyopathies: an American Heart Association Scientific Statement from the Council on Clinical Cardiology, Heart Failure and Transplantation Committee; Quality of Care and Outcomes Research and Functional Genomics and Translational Biology Interdisciplinary Working Groups; and Council on Epidemiology and Prevention.. Circulation.

[37] Kushwaha SS, Fallon JT, Fuster V (1997). Restrictive cardiomyopathy.. N Engl J Med.

[38] Davis J, Wen H, Edwards T, Metzger JM (2007). Thin filament disinhibition by restrictive cardiomyopathy mutant R193H troponin I induces Ca2+-independent mechanical tone and acute myocyte remodeling.. Circ Res.

[39] Rarick HM, Tu XH, Solaro RJ, Martin AF (1997). The C terminus of cardiac troponin I is essential for full inhibitory activity and Ca2+ sensitivity of rat myofibrils.. Journal of Biological Chemistry.

[40] Kass DA, Solaro RJ (2006). Mechanisms and use of calcium-sensitizing agents in the failing heart.. Circulation.

